# Sleep Disturbance as a Potential Modifiable Risk Factor for Alzheimer’s Disease

**DOI:** 10.3390/ijms20040803

**Published:** 2019-02-13

**Authors:** Eiko N. Minakawa, Keiji Wada, Yoshitaka Nagai

**Affiliations:** 1 Department of Degenerative Neurological Diseases, National Institute of Neuroscience, National Center of Neurology and Psychiatry, Kodaira 187-8502, Japan; wada@ncnp.go.jp; 2 Department of Neurotherapeutics, Osaka University Graduate School of Medicine, Osaka 565-0871, Japan

**Keywords:** Alzheimer’s disease, sleep disturbance, sleep fragmentation, slow-wave sleep, amyloid beta, tau, proteostasis, default-mode network, cognitive behavioral therapy for insomnia

## Abstract

Sleep disturbance is a common symptom in patients with various neurodegenerative diseases, including Alzheimer’s disease (AD), and it can manifest in the early stages of the disease. Impaired sleep in patients with AD has been attributed to AD pathology that affects brain regions regulating the sleep–wake or circadian rhythm. However, recent epidemiological and experimental studies have demonstrated an association between impaired sleep and an increased risk of AD. These studies have led to the idea of a bidirectional relationship between AD and impaired sleep; in addition to the conventional concept that impaired sleep is a consequence of AD pathology, various evidence strongly suggests that impaired sleep is a risk factor for the initiation and progression of AD. Despite this recent progress, much remains to be elucidated in order to establish the benefit of therapeutic interventions against impaired sleep to prevent or alleviate the disease course of AD. In this review, we provide an overview of previous studies that have linked AD and sleep. We then highlight the studies that have tested the causal relationship between impaired sleep and AD and will discuss the molecular and cellular mechanisms underlying this link. We also propose future works that will aid the development of a novel disease-modifying therapy and prevention of AD via targeting impaired sleep through non-pharmacological and pharmacological interventions.

## 1. Introduction

**Figure 1:**
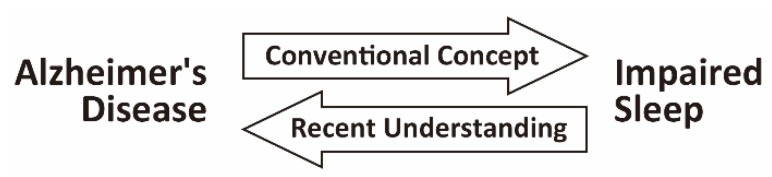
Bidirectional relationship between Alzheimer’s disease (AD) and impaired sleep. Impaired sleep is prevalent in patients with AD. Both epidemiological and experimental studies have led to the recent concept of a bidirectional relationship between AD and impaired sleep. In addition to the conventional concept that impaired sleep is a consequence of AD pathology affecting brain regions regulating the sleep–wake or circadian rhythm, impaired sleep has been suggested as a risk factor for the initiation and progression of AD.

Sleep disturbance is a common symptom associated with Alzheimer’s disease (AD), which is the leading cause of dementia worldwide [1]. More than 60% of patients with AD develop sleep disturbance, which often occurs at the early stages of the disease or even before the onset of major cognitive decline [2]. Impaired sleep in these patients has been attributed to the progression of AD pathology to brain regions that regulate the sleep–wake or circadian rhythm (Figure 1) [3]. However, various epidemiological studies have demonstrated the association between impaired sleep and an increased risk of AD or AD-related pathology [3]. Multiple studies using animal models of AD have also indicated that impaired sleep exacerbates memory decline and AD-related pathology (Figure 1) [4]. These recent findings suggest that sleep disturbance is a potential modifiable risk factor for AD and could be a novel target for disease-modifying therapies to prevent the development of AD and/or ameliorate the cognitive decline in patients with AD [3]. In this review, we will first provide an overview on the epidemiological and experimental studies that have linked AD and sleep. We will then describe experimental studies that have examined the causal relationship between impaired sleep and AD, and will discuss the molecular and cellular mechanisms that might underlie this link. Finally, we will propose future research directions, including the establishment of a novel disease-modifying therapy and the prevention of AD via targeting impaired sleep in patients with AD and cognitively normal people.

## 2. Age-Related Sleep Alterations

Physiological sleep in mammals, including humans, is composed of rapid eye movement (REM) sleep and non-REM (NREM) sleep. Human NREM sleep can be classified into three stages according to its depth, namely stage N1, N2, and N3, using electroencephalogram (EEG) findings that are characteristic of each stage [5]. Stage N3, the deepest NREM sleep, is characterized by a dominant EEG activity that consists of high-voltage slow waves with a frequency range of 1–4 Hz and is thus referred to as slow-wave sleep (SWS) [6].

The age-associated alterations in sleep architecture have been well characterized. The most prominent changes are increased sleep fragmentation by intermittent nocturnal arousals and a reduced amount of SWS, which is associated with shorter overall sleep duration and increased N1 and N2 duration [7]. Compared with these changes in NREM sleep, REM sleep is relatively spared except for a decreased REM latency with age [7] until around 80 years old, after which its duration is also reduced [8].

## 3. Sleep Disturbance in Alzheimer’s Disease (AD)

### 3.1. Sleep Abnormalities in Patients with Alzheimer’s Disease (AD)

Patients with AD often experience difficulty falling asleep, repeated nocturnal arousals, early arousals in the morning, and excessive sleepiness during daytime [9]. One or more sleep disorders, including insomnia, circadian rhythm sleep–wake disorders, sleep-related breathing disorders (SRBD), and sleep-related movement disorders, underlie these symptoms [10].

The most consistently reported changes in sleep architecture in patients with mild to moderate AD are sleep fragmentation, which is due to an increased number and duration of intermittent nocturnal arousals, a reduced amount of SWS, a resulting decrease in overall sleep duration, and an increase in N1 [11]. These AD-associated changes in NREM sleep seem to be an exaggeration of sleep alterations that are associated with normal aging, which become more pronounced with an increase in the severity of AD [12]. In addition, sleep spindles and K complexes, which are the EEG markers of stage N2, exhibit poorer formation, lower amplitude, shorter duration and smaller number [11]. These changes are mostly similar to the age-associated change in these stage N2 markers, except for the spindle formation, whose age-related change is still controversial [13]. Meanwhile, the total duration of REM sleep, which is relatively spared in normal aging, is reduced in patients with AD due to a reduced duration of each REM episode [14]. Other REM sleep variables, such as the number of REM episodes and REM latency, are usually spared in AD [14].

The alterations in the diurnal rhythm of activity and sleep due to circadian rhythm dysregulation are also present in patients with preclinical AD and symptomatic AD [15]. A disrupted circadian rhythm can cause sundowning syndrome or nocturnal delirium [16], in which patients often become agitated, restless or anxious in the early evening. These symptoms usually resolve during the daytime, but greatly impair the quality of life of patients, families and caregivers [16].

### 3.2. Sleep Disturbance as a Consequence of AD Pathology

Sleep alterations in patients with AD have been interpreted as a consequence of the progression of AD pathology to brain regions that are involved in the regulation of the sleep–wake or circadian rhythm [3]. AD pathology affects galaninergic neurons in the intermediate nucleus of the hypothalamus [17]. This area is a homolog of the ventrolateral preoptic nucleus of rodents, which is selectively active during sleep [18] and sends inhibitory projections to wake-promoting areas [19]. The number of remaining galaninergic neurons in the intermediate nucleus of autopsied AD brains has been found to be negatively correlated with the severity of ante-mortem sleep fragmentation evaluated within one year of death by actigraphy [17], which suggests that galaninergic neuronal loss due to AD pathology leads to sleep fragmentation in patients with AD.

AD pathology also affects the cholinergic neuronal network [14], which comprises the brainstem, thalamus, basal forebrain and cerebral cortex. This network regulates the initiation and maintenance of REM sleep [14]. The primary circadian pacemaker in the mammalian brain is the hypothalamic suprachiasmatic nucleus (SCN), which is also affected by AD pathology. AD is associated with a significant loss of vasopressin- and vasoactive intestinal peptide-expressing neurons, which are involved in the maintenance of circadian function in the SCN [20,21,22].

Various transgenic or knock-in mouse models of AD also develop sleep abnormalities, such as increased wakefulness [23,24,25], a decrease in NREM sleep [23,25] and REM sleep [23], circadian rhythm delay [24], or a reduced amplitude of the circadian rhythm [25]. The sleep–wake patterns of APPswe/PS1dE9 transgenic mice [26] are normal before Aβ deposition, but a tendency of increased wakefulness and decreased sleep starts at the age when Aβ deposition is initially observed. Furthermore, these sleep abnormalities exacerbte with age and increased Aβ deposition [23]. Furthermore, APPswe/PS1dE9 mice that are actively immunized with Aβ, which decreases Aβ deposition in the brain, showed a normal sleep–wake pattern [23]. Taken together, these findings suggest that sleep disturbance is caused not only by a neuronal loss in the brain regions regulating sleep or circadian rhythm, but also by Aβ accumulation in the brain.

## 4. Sleep Disturbance as a Risk Factor of AD

### 4.1. Epidemiological Studies

Contrary to the conventional understanding that impaired sleep in patients with AD is a consequence of AD-related pathology, multiple recent epidemiological studies have suggested that sleep disturbance could be a risk factor for cognitive decline and AD. According to a recent meta-analysis, sleep disturbance or sleep disorders, including short or long sleep duration, poor sleep quality (difficulty in falling asleep or increased intermittent nocturnal arousal), circadian rhythm abnormality, insomnia or SRBD, were associated with a significant increase in the risk ratio (RR) for cognitive impairment (RR: 1.64, 95% CI: 1.45–1.87), preclinical AD (RR: 3.78, 95% CI: 2.27–6.30) and AD diagnoses based on the ICD-9 (International Classification of Diseases, Ninth edition) or DSM-IV (Diagnostic Statistical Manual, Fourth edition) (RR: 1.55, 95% CI: 1.25–1.93) [27]. 

In a prospective study that used actigraphy to quantitatively assess the sleep of 737 community-dwelling older adults without dementia, a higher level of sleep fragmentation due to increased intermittent nocturnal arousal was associated with an increased risk of AD (hazard ratio = 1.22, 95% CI: 1.03–1.44) [28]. Individuals with high sleep fragmentation (in the 90th percentile) at baseline had a 1.5-fold higher risk of developing AD compared to those with low sleep fragmentation (in the 10th percentile) during the 6-year follow-up period (mean = 3.3 years) [28]. In addition, in a positron emission tomography (PET) study that examined the association between sleep variables and amyloid beta (Aβ) deposition in older people without dementia, a self-reported shorter sleep duration and poorer sleep quality were associated with significantly greater in vivo Aβ deposition in the precuneus [29], which is affected by Aβ pathology in preclinical AD [30].

Although these studies indicate an association between impaired sleep and AD, epidemiological observational studies conducted so far are limited in discerning the causal relationship between impaired sleep and AD, especially considering the relatively short follow-up periods compared to the long disease course of AD [3]. For example, a subgroup meta-analysis for the effect of sleep disturbance demonstrated that both short and long sleep duration were associated with a higher risk of cognitive decline or AD [27]. Additional studies are needed to determine whether both short and long sleep duration do indeed affect the disease course of AD, or whether either of these is a prodromal symptom of AD or reflecting the comorbidities of AD, such as depression.

### 4.2. The Causal Relationship between Sleep Disturbance and AD Pathology

Various animal models of AD and sleep disturbance have been used to assess the causal relationship between sleep disturbance and AD and the molecular or cellular mechanisms potentially underlying this link. Kang et al. (2009) were the first to report that chronic sleep restriction accelerates Aβ deposition in the brain using two transgenic AD mouse models (APPswe and APPswe/PS1dE9 mice) [31]. Other studies have also demonstrated that sleep deprivation or restriction in various AD models exacerbates AD-related biochemical or pathological changes in mice brains, such as an increase in Aβ or phosphorylated tau [32,33], an increase in insoluble phosphorylated tau and glial fibrillary acidic protein levels [34], and an increase in Aβ_40_, Aβ_42_ and β-site amyloid-precursor-protein-cleaving enzyme 1 (BACE1), which produce toxic Aβ species [35].

In these studies, sleep disturbance was induced in the mice either by intermittent gentle tactile stimuli, resulting in total deprivation of sleep [31], by the platform-over-water technique, resulting in elimination of REM sleep and a decrease in SWS [31,32,33,35], or by alteration of the light–dark cycle, resulting in a disrupted circadian rhythm [34]. The limitation of these studies is that the resultant sleep–wake patterns using the above methods are different from those observed in patients with AD or in normal aging. In addition, these methods induce relatively high levels of stress in mice. These acute or chronic behavioral stresses could aggravate the AD pathology via an increase in Aβ [36] and might therefore be a confounding factor. In a recent study, we took advantage of a novel device that induces impaired sleep closely resembling that of patients with AD (i.e., an increase in sleep fragmentation and a decrease in the amount of SWS) without severe stress [37] and found that chronic sleep fragmentation indeed aggravates Aβ deposition in the AD mice brain [38]. Notably, the severity of Aβ deposition showed a significant positive correlation with the severity of sleep fragmentation [38]. Since all mice were subjected to sleep impairment by a unified protocol, our results strongly suggest that the aggravation of Aβ pathology is more directly related to sleep impairment than the behavioral stress, if any, that was induced by the device we used to induce sleep impairment. Considering this point, our results are consistent with a previous epidemiological study that demonstrated an association between sleep fragmentation and an increased risk of AD [28]. Thus, our evidence supports the view that sleep disturbance in older people and patients with AD affects the disease course of AD.

## 5. Molecular/Cellular Mechanisms that Link AD and Sleep

### 5.1. Impaired Sleep Alters the Dynamics of Aβ and Tau in the Brain

The two major pathological hallmarks of AD are senile plaques, which are the extracellular deposits that are mainly composed of insoluble Aβ, and neurofibrillary tangles (NFT), which are the intracytoplasmic deposits that are mainly composed of hyperphosphorylated insoluble tau [39]. The dynamics of extracellular Aβ in relation to neuronal activity and the sleep–wake cycle have been extensively studied using various in vitro and in vivo animal and humans. Several recent studies have also examined the relationship between the dynamics of tau and sleep.

Extracellular Aβ in the central nervous system can be detected as a soluble form in cerebrospinal fluid (CSF) in humans as well as in CSF or interstitial fluid (ISF) in mice. The soluble Aβ shows diurnal fluctuation in both healthy young humans and mice, with an increase during wakefulness and a decrease during sleep [31,40]. The amplitude of this diurnal fluctuation is decreased in the CSF of older people without Aβ deposition and disappears in older people with Aβ deposition [40]. In APPswe/PS1dE9 mice, the diurnal Aβ fluctuation in ISF disappears when mice develop Aβ deposition [23]. These studies suggest that the dynamics of extracellular soluble Aβ is one of the potential mechanisms linking sleep and an increased risk of AD. Therefore, the mechanisms that affect the dynamics of soluble Aβ in ISF or CSF have been extensively studied.

Various studies have confirmed that Aβ production is regulated by neuronal action potential firing. An increase in neuronal firing leads to an increase in the extracellular secretion of soluble Aβ in an activity-dependent manner [41,42]. In vivo experiments have also demonstrated a direct relationship between increased neuronal activity and increased production of extracellular soluble Aβ in the brain, which was detected in the interstitial fluid (ISF) [43]. Furthermore, a sustained increase in the neuronal activity by optogenetic stimulation induces an increase in soluble Aβ in ISF followed by insoluble Aβ deposition in the projection area of the stimulated neurons [44]. Consistent with these studies, extended wakefulness by total sleep deprivation results in an increased level of soluble Aβ in the ISF or CSF [31,45]. Interestingly, specific disruption of SWS, but not sleep duration or sleep efficiency, induces an increase in CSF Aβ [46]. This suggests that each sleep component may influence the dynamics of extracellular Aβ in different ways.

The mechanism underlying the decrease in soluble Aβ during sleep is still controversial. The interchanging convective flow of ISF and CSF in the interstitial space of the brain has been reported to play a crucial role in the removal of the extracellular metabolites, including Aβ, in the brain [47]. Furthermore, Xie et al. reported that natural sleep is associated with a 60% increase in the interstitial space in the brain, which results in an increase in the clearance efficiency of interstitial metabolites, including Aβ, by the increased convective flow of CSF and ISF [48]. The removal of interstitial Aβ by this clearance system, named the glymphatic system, may be one of the mechanisms underlying the decrease in CSF and ISF Aβ during sleep. Meanwhile, recent human studies have analyzed Aβ turnover in CSF by radioactive labeling of Aβ [49,50]. These studies concluded that a decreased production of Aβ due to reduced neuronal activity rather than the increased clearance of Aβ is a necessary and critical factor for the decrease in CSF Aβ during sleep [49,50].

Extracellular soluble tau is another important component in ISF and CSF that is related to AD pathology, while intracellular aggregated tau is a pathological hallmark of AD. Recent studies have indicated that the total tau and phosphorylated tau in CSF are biomarkers that differentiate patients with AD from healthy controls as well as those with mild cognitive impairments due to preclinical AD from those due to other conditions [51,52]. 

Similarly to Aβ, neuronal activity has been found to induce the extracellular release of tau in an in vitro model [53]. Neuronal activity also induces the propagation of aggregated tau pathology in vivo via the extracellular release of tau and uptake of released tau by nearby neurons [54]. Extracellularly released tau is indeed detectable in the ISF of tau transgenic mouse models [55,56,57,58]. Multiple recent studies have examined the in vivo dynamics of the extracellular tau in ISF and CSF in relation to neuronal activity and the sleep–wake cycle. In tau transgenic mice with regulatable expression, the half-life of extracellular soluble ISF tau was revealed to be 17.3 days [56]. This is remarkably longer than that of Aβ, which shows diurnal fluctuation. Consistent with this finding, poorer sleep quality, which was measured for six consecutive nights before CSF collection, was found to have a significant negative correlation with an increase in CSF tau, while acute deprivation of SWS did not lead to CSF tau elevation [46]. Meanwhile, a very recent study demonstrated that acute sleep deprivation leads to a remarkable increase of tau in both mice ISF and human CSF [59]. Importantly, another recent study that used a combination of sleep monitoring by single-channel EEG with PET imaging and CSF analysis of both Aβ and tau revealed that a decrease in SWS, especially at the lowest frequencies of 1–2 Hz, was more associated with the accumulation of tau than that of Aβ [60].

Together, these studies suggest that impaired sleep affects the dynamics of both Aβ and tau, which may lead to the exacerbation of AD-related pathology. Further studies are awaitedto determine whether the dynamics of Aβ and tau are regulated via same mechanisms of production and clearance and via similar components of sleep.

### 5.2. Prolonged Wakefulness Induces Impaired Proteostasis, a Common Pathomechanism Underlying Neurodegenerative Diseases

**Figure 2:**
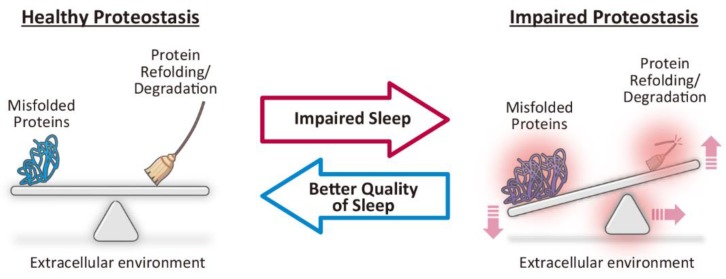
Impaired sleep as a potential therapeutic target to restore proteostasis. Healthy proteostasis is maintained through the coordination of various intra- and extracellular systems that regulate protein synthesis, folding, disaggregation, and degradation (left). Increased synthesis of misfolded proteins, dysfunction of protein refolding or degradation systems, or changes in extracellular environment can lead to impaired proteostasis and result in the accumulation of misfolded and aggregation-prone toxic proteins (right), which is a common pathomechanism underlying neurodegenerative diseases. Based on recent studies that have indicated that impaired sleep leads to impaired proteostasis (middle; red arrow), future studies that better examine the relationship between sleep and proteostasis could lead to the development of novel therapeutics that restore healthy proteostasis via better quality of sleep (middle; blue arrow).

Proteins with proper functions are indispensable for living organisms. Intracellular and in vivo protein quality is maintained in a homeostatic manner through the coordination of multiple intra- and extracellular systems that regulate protein synthesis, folding, disaggregation, and degradation [61]. The resultant homeostasis of protein quality (Figure 2; left), which is called proteostasis, is of general importance for maintaining human health. 

Impaired proteostasis (Figure 2; right) is a common pathomechanism underlying neurodegenerative diseases, such as AD, Parkinson’s disease (PD), dementia with Lewy bodies (DLB), amyotrophic lateral sclerosis and Huntington’s disease [39]. Neurodegenerative diseases are characterized by selective and progressive neuronal degeneration, which is accompanied by abnormal protein aggregates in the regions of the central nervous system (CNS) that are characteristic of each disease [39]. Patients exhibit slowly progressive neurological or psychiatric symptoms of various types, such as cognitive or motor impairment, or involuntary movements, depending on the affected regions specific to each disease. Recent studies have reported that impaired proteostasis and the resultant accumulation of misfolded and aggregation-prone proteins (Figure 2; right) exhibit neurotoxicity and lead to neuronal dysfunction followed by neurodegeneration [61].

Sleep affects proteostasis in the brain. A detailed transcriptomic study revealed that the most abundant categories of genes that are upregulated in the mice brain during sleep are those involved in macromolecule biosynthesis, such as structural components of ribosomes, translation initiation and elongation factors and tRNA activators [62]. In addition, genes involved in intracellular transport, such as vesicle-mediated protein trafficking, are also upregulated during sleep [62]. 

Among the multiple molecules/pathways involved in the refolding or degradation of misfolded proteins to maintain proteostasis, such as chaperones, the ubiquitin–proteasome system and autophagy, the relationship between sleep and the unfolded protein response (UPR) pathway has been studied in detail. Prolonged wakefulness by sleep deprivation activates the UPR pathway, which is one of the major mechanisms that prevent the accumulation of misfolded proteins and maintains proteostasis [63]. When the endoplasmic reticulum (ER), a major site of protein folding and post-translational modification, is overloaded and stressed by the accumulation of misfolded and potentially toxic proteins, the UPR is activated and triggers different levels of downstream pathways according to the duration and the severity of the ER stress [64]. Mild or transient ER stress induces adaptive or protective pathways, such as increased transcription of chaperones for proper protein refolding, attenuation of general protein translation and removal of misfolded proteins for degradation at the proteasome. When ER stress is not alleviated by these pathways, the pro-apoptotic signaling pathway is activated, which leads to cellular injury or cell death [64]. Various studies have shown that prolonged wakefulness by sleep deprivation for six hours or longer leads to the upregulation of protective or adaptive pathways downstream of UPR activation in the rodent brain, such as the increased production of BiP/GRP78, a major ER chaperone and a marker of UPR activation [63,65,66,67]. However, in the aged mice brain, six hours of sleep deprivation failed to induce the protective pathways downstream of UPR activation, such as the upregulation of BiP/GRP78 or inhibition of general protein translation. On the contrary, six hours of sleep deprivation did activate pro-apoptotic signaling pathways [68]. 

These studies suggest that prolonged wakefulness by acute sleep deprivation is sufficient to at least transiently impair proteostasis in the brain (Figure 2, middle; red arrow), and that aging impairs the protective responses against impaired sleep, which could in turn lead to neurodegeneration. Further studies on the role of sleep in the maintenance of proteostasis via the UPR and other pathways could aid the development of novel therapeutics that can restore healthy proteostasis via better quality of sleep and could represent disease modification strategies for neurodegenerative diseases (Figure 2, middle; blue arrow).

### 5.3. Impaired Sleep May Aggravate the Propagation of AD-Related Pathology via Impaired Functional Connectivity in the Brain

Functional connectivity in the brain is defined as inter-regional correlations in the neuronal activation patterns of anatomically separate brain regions [69]. Functional connectivity reflects the integrity of communication between two functionally related brain regions [70]. Independent component analysis of the functional connectivity at the resting state, when individuals are awake but not focused on their external environment, has identified several functional resting-state networks (RSNs) in the cerebral cortex that exhibit increased activity, specifically at resting state [70]. The default mode network (DMN) is one of the major RSNs and underlies most of the baseline brain activity at rest [71]. The core regions of the DMN include the medial prefrontal cortex, posterior cingulate cortex, precuneus and parietal cortex, all of which have structural interconnections and functional connectivity [72]. 

Intriguingly, a recent study demonstrated that functional connectivity in the brain shows a diurnal patternand that nocturnal sleep restores morning-to-evening connectivity changes [73]. A lack of sleep has been associated with a deficit in the recovery of functional connectivity on the following morning within various networks, including the DMN [73]. Another study demonstrated that the significant functional correlations between frontal and posterior areas of the DMN become non-significant during SWS, which suggests that the integrity of the DMN is decreased during deep sleep [74]. These studies indicate the potential importance of sleep on the maintenance of the DMN during arousal. 

It has also been well established that all regions of the DMN are vulnerable to AD-related pathology [75]. Indeed, DMN impairment is present in early symptomatic AD and progresses with the disease course [76]. DMN impairment is even observed in preclinical AD, when AD-related histopathology accumulates before overt clinical symptoms appear [76]. Furthermore, the carriers of the *ApoE ε4* allele, which is the most potent risk factor for AD, also show DMN impairment similar to that of preclinical AD, even in the absence of Aβ deposition in the brain [77].

The precise mechanism underlying the relationship between the DMN and the progression of AD pathology has yet to be fully elucidated. However, recent studies have strongly suggested that misfolded neurotoxic proteins, such as Aβ and tau in the case of AD, are transmitted along interconnected neural networks [78,79]. Consistent with this, it is plausible that misfolded toxic proteins can be propagated from the brain regions that are initially affected by AD pathology to adjacent healthy brain regions [76]. This protein propagation via interconnected brain regions could eventually lead to the gradual deterioration of the entire brain network from a semi-functional state to a dysfunctional state as misfolded proteins accumulate over the years [76]. From this point of view, the alterations in functional connectivity due to impaired sleep, especially at the preclinical or early stages of AD, might be an additional pathomechanism underlying the progression of AD-related pathology that results from impaired sleep.

### 5.4. Other Mechanisms that May Link Impaired Sleep and AD-Related Pathology

Inflammatory immune responses, blood–brain barrier (BBB) disruption, and oxidative stress are known to affect AD-related pathology, which can also be induced by impaired sleep [3,80].

Acute and chronic sleep loss in humans result in the induction of both cellular and humoral immunological responses. An increase in the number of circulating leukocytes (mainly monocytes and neutrophils) and increased levels of proinflammatory cytokines, such as interleukin-1β (IL-1β) and IL-6 and tumor necrosis factor-α (TNF-α), are observed after acute sleep deprivation or subacute sleep restriction [81]. The resulting low-grade systemic inflammation could facilitate neuroinflammation when sleep impairment is sustained, which could aggravate AD-related brain pathology [82]. Indeed, chronic sleep loss in rodents has been associated with microglial activation and astrocytic phagocytosis in the brain [83]. In addition, chronic low-grade inflammation has been proposed to underlie the BBB breakdown following sleep loss observed in rodent models [84], which could also worsen AD-related pathology [85]. Furthermore, sleep deprivation promotes oxidative stress in the rodent brain [86]. A recent prospective epidemiological study indicated that obstructive sleep apnea (OSA) in cognitively normal older people is associated with increased Aβ deposition [87]. Besides the sleep fragmentation itself due to OSA, which could affect Aβ dynamics (as discussed in Section 5.1), a combination of hypoxemia, neuroinflammation, and oxidative stress could be additional mechanisms underlying the exacerbation of AD pathology in patients with OSA.

## 6. Conclusions and Future Directions

Impaired sleep is prevalent in patients with AD, which often occurs in the early or even preclinical stages of AD. Both epidemiological and experimental studies have led to the recent concept of a bidirectional relationship between AD and impaired sleep (Figure 1). In addition to the conventional concept that impaired sleep is a consequence of AD-related pathology, impaired sleep has been suggested to be a risk factor for the initiation and progression of AD, at least in cognitively normal older people and in patients with AD. Despite this recent progress, much remains to be elucidated in future works that will aid the development of therapeutic interventions against impaired sleep to prevent or alleviate the disease course of AD. 

First, the essential components of “better sleep” that reduce the risk for AD need to be determined. A recent study demonstrated that an acute inhibition of SWS is sufficient to affect Aβ dynamics in humans [46]. While the importance of REM sleep in regulating NREM sleep has been established [88], additional studies are crucial in obtaining a more comprehensive understanding of the roles and interactions between the different components of sleep, including REM sleep, light NREM sleep and SWS. Furthermore, the molecular and cellular mechanisms underlying the link between AD and these different components of sleep remain to be determined. It would also be necessary to determine the contribution of other sleep-related factors to AD-related pathology, such as the optimal duration of sleep that reduces the risk of AD.

Second, potential therapeutic methods to achieve “better sleep” need to be investigated. Recent meta-analyses have demonstrated the effect of non-pharmacological treatment by cognitive behavioral therapy for insomnia (CBT-I) on primary chronic insomnia [89,90]. In addition, several randomized control studies have shown that CBT-I is more effective than pharmacotherapy using conventional hypnotics that target γ-aminobutyric acid (GABA)_A_ receptor-mediated systems [91]. CBT-I provided via cost-effective and accessible ways, such as computerized and online platforms or video conferencing, has also shown therapeutic benefits [91]. While these non-pharmacologic methods are recommended as first-line treatments for primary chronic insomnia [92], the recent development of novel hypnotics with different mechanisms of action and potentially better safety, especially in elderly patients, might provide better therapeutic opportunities compared to traditional hypnotics [93]. Whether these non-pharmacological and pharmacological treatments can also achieve “better sleep” that reduces the risk for AD development and progression remains to be determined.

Furthermore, chronic short sleep is highly prevalent in both healthy young adults and adolescents, especially in developed countries [94]. These people generally have insufficient sleep during weekdays and use weekends to catch up on sleep, which leads to the subjective normalization of sleepiness. However, several studies have demonstrated that weekend sleep is not sufficient to fully recover the cognitive performance deficit induced by sleep insufficiency during weekdays [95,96,97]. Whether the accumulation of sleep insufficiency that begins from adolescence or young adulthood affects the molecular or cellular links between sleep and AD and whether this could lead to an increased risk of AD development would be particularly important for the primary prevention of AD.

Last but not least, impaired sleep mainly due to sleep fragmentation and a decrease in SWS is also prevalent in patients with various neurodegenerative diseases other than AD. Considering that neurodegenerative diseases, including AD, share a common pathomechanism of misfolded protein accumulation and impaired proteostasis, “better sleep” that reduces the risk for AD might also alleviate the disease course of other neurodegenerative diseases. Elucidating the link between impaired sleep and the dynamics of misfolded proteins that accumulate in each disease, such as α-synuclein in PD and DLB as well as Aβ and tau in AD, could lead to the development of a novel disease-modifying therapy that has far-reaching implications for neurodegenerative diseases in general.

## Abbreviations

AD	Alzheimer’s disease
REM	Rapid Eye Movement
NREM	Non-Rapid Eye Movement
EEG	Electroencephalogram
SWS	Slow-wave Sleep
SRBD	Sleep-related breathing disorders
SCN	Suprachiasmatic Nucleus
RR	Risk Ratio
ICD-9	International Classification of Diseases, Ninth Edition
DSM-IV	Diagnostic Statistical Manual, Fourth Edition
Aβ	Amyloid Aβ
BACE1	β-site amyloid precursor protein cleaving enzyme 1
NFT	Neurofibrillary Tangles
CSF	Cerebrospinal Fluid
ISF	Interstitial Fluid
PD	Parkinson’s Disease
DLB	Dementia with Lewy Bodies
CNS	Central Nervous System
UPR	Unfolded Protein Response
ER	Endoplasmic Reticulum
RSN	Resting State Network
DMN	Default Mode Network
TNF-α	Tumor Necrosis Factor-α
OSA	Obstructive Sleep Apnea
CBT-I	Cognitive Behavioral Therapy for Insomnia
GABA	γ-aminobutyric Acid
